# Functional interplay between PPM1G and the transcription elongation machinery

**Published:** 2016-03-14

**Authors:** Swapna Aravind Gudipaty, Iván D’Orso

**Affiliations:** 1 Huntsman Cancer Institute, Salt Lake City, UT 84112, USA; 2 Department of Microbiology, The University of Texas Southwestern Medical Center, Dallas, TX 75390, USA

**Keywords:** NF-κB, DNA damage signaling, phosphatase, PPM1G/PP2Cγ, P-TEFb, 7SK snRNP, RNA polymerase II, transcription elongation

## Abstract

Transcription elongation is a critical regulatory step in the gene expression cycle. One key regulator of the switch between transcription initiation and elongation is the P-TEFb kinase, which phosphorylates RNA polymerase II (Pol II) and several negative elongation factors to relieve the elongation block at paused promoters to facilitate productive elongation. Here, we highlight recent findings signifying the role of the PPM1G/PP2Cγ phosphatase in activating and maintaining the active transcription elongation state by regulating the availability of P-TEFb and blocking its assembly into the catalytic inactive 7SK small nuclear ribonucleoprotein (snRNP) complex.

## Introduction

Studies over the past three decades have provided insights into the importance of transcription regulatory mechanisms in metazoan cells. RNA polymerase II (Pol II) activity can be regulated at several steps of the transcription cycle including pre-initiation, DNA melting, initiation, promoter clearance, and elongation ^[1–7]^. It has, more recently, became well-established in the field that transcription elongation control is vital for the regulation of several key biological processes including response to environmental cues, cell fate choice, differentiation, and development. Transcription elongation is precisely regulated by the action of two sets of elongation factors (negative and positive) that dictate transcriptional pausing and pause release, respectively. Sequence-specific transcription factors utilize co-regulators (co-activators and co-repressors), large protein complexes carrying enzymatic activities, to precisely modulate one or more steps in the transcription cycle. Great progress has been made in identifying several transcriptional co-regulators and in describing their critical importance for Pol II transcription such as acetyl transferases, deacetylases, kinases and phosphatases, among others ^[8]^. Here, we discuss the discovery of a novel transcriptional co-regulator, mechanistic insights into its role in promoting transcription elongation and future challenges.

## Transcriptional pausing

Promoter-proximal Pol II pausing is a key rate-limiting and highly regulated step in the transcription cycle. Genome-wide analysis in metazoan cells showed that the major form of Pol II is bound to promoter-proximal regions and poised for rapid entry into productive elongation ^[9–12]^. Notably, Pol II pausing is prevalent at genes involved in development and response to stimuli, suggesting that transcriptional pausing during early elongation plays important, variable, and regulated rate-limiting roles in the rapid and precise control of gene expression ^[13,14]^.

**Figure 1:**
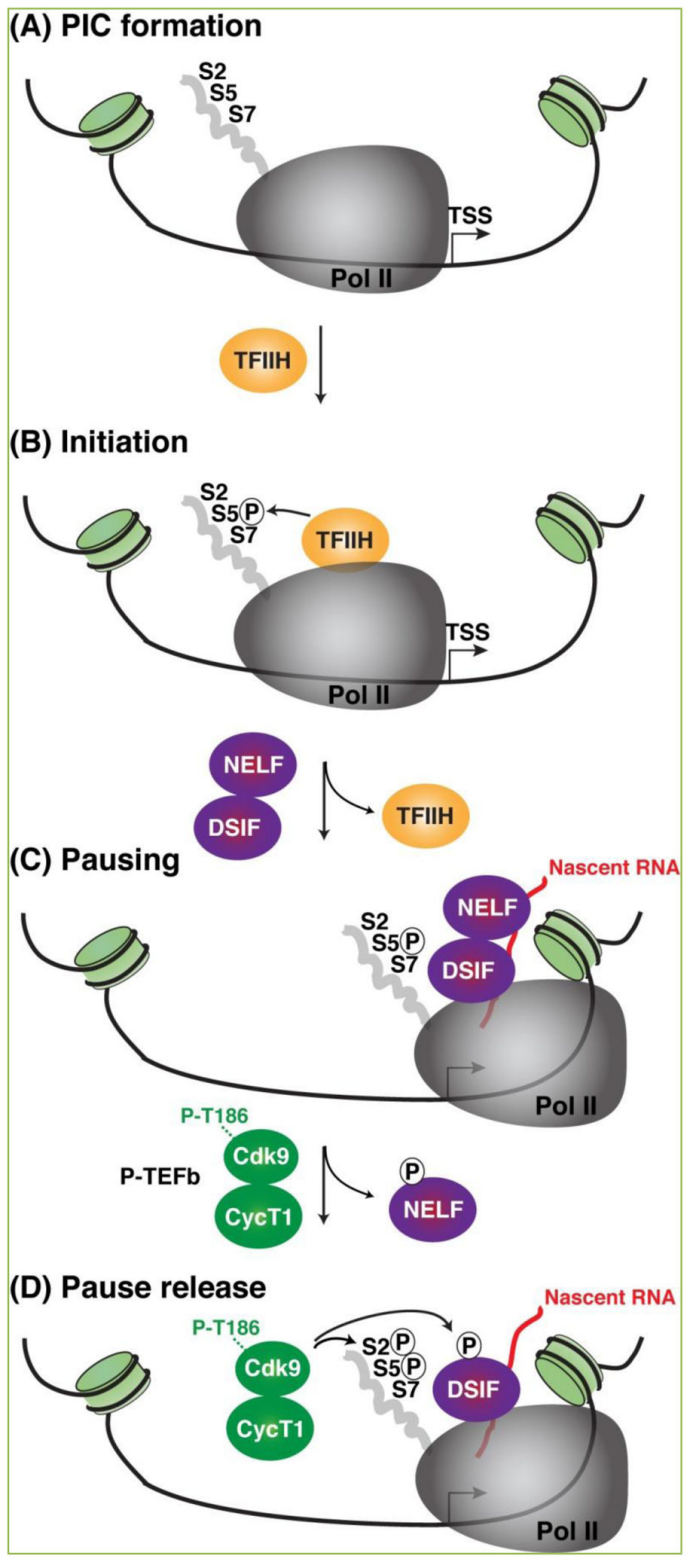
Transcription steps controlling Pol II pausing and pause release **A.** The transcription pre-initiation complex (PIC), composed of Pol II and additional general transcription factors (not shown for simplicity), assemble on the template DNA before the transcription start site (TSS). The largest subunit of Pol II contains a C-terminal domain (CTD) composed of 52 heptad repeats with the consensus sequence YSPTSPS where Ser2 (S2), Ser5 (S5) and Ser7 (S7) serve as acceptor sites for multiple kinases. **B.** The TFIIH complex (containing the Cdk7 kinase) phosphorylates Ser5 residues on the CTD (S5P) to facilitate initiation and promoter clearance. **C.** Upon synthesis of a short RNA chain (>18 nt-long, shown in red), TFIIH is evicted and the negative elongation factors DSIF and NELF are recruited through cooperative interactions with nascent RNA and Pol II to promote pausing. **D.** P-TEFb is recruited to Pol II and phosphorylates NELF (which is evicted from the DSIF-Pol II complex) in addition to DSIF and Pol II, which together facilitate Pol II pause release.

After transcription initiation and promoter clearance due to the action of the TFIIH kinase, Pol II pauses between the transcription start site (TSS) and the +1 nucleosome after transcribing a short (~20–60 nt) RNA chain by the action of negative elongation factors (Figure 1). There are two major negative elongation factors: 5,6-dichloro-1-β-D-ribofuranosyl-benzimidazole (DRB) sensitivity-inducing factor (DSIF) and Negative Elongation Factor (NELF), which interact with early elongation complexes to halt elongation and induce transcriptional pausing. Most metazoan genes require cooperative inhibition by DSIF and NELF for robust Pol II pausing and gene activation ^[15]^. Interestingly, levels of DSIF and NELF at promoter-proximal regions directly correlate with total Pol II occupancy levels, suggesting that these factors associate with most early elongation complexes ^[4]^, and at most Pol II regulated genes thus implying a general role in controlling pausing ^[9, 10, 12]^.

DSIF is composed of two subunits (p160 and p14), which are the human homologues of *Saccharomyces cerevisiae* Spt5/Spt4 factors. DSIF was first identified by Wada *et al*. using HeLa cell nuclear extracts for factors that cause pausing of Pol II in conjunction with the transcription inhibitor DRB ^[16–18]^. In addition to its negative function in elongation, Wada *et al*. found that DSIF associated with Pol II and stimulated the rate of elongation by Pol II in vitro in a reaction containing limiting nucleotides ^[16, 19]^.

Similarly, using an elegant biochemical approach, Yamaguchi *et al*. described the identification and purification from HeLa nuclear extracts of a factor required for DRB-sensitive transcription and they referred to it as NELF ^[20]^. NELF is composed of five polypeptides (NELF-A, -B, -C/D and -E), the smallest (NELF-E) of which is identical to the RNA-binding protein RD, and whose RNA-binding activity is critical for NELF function as negative elongation factor ^[21]^. Upon NELF recruitment to Pol II at promoter-proximal regions, NELF inhibits Pol II activity by binding the nascent RNA chain and preventing further elongation ^[15,20]^.

While DSIF binds Pol II directly and stably without affecting its polymerase activity, NELF does not bind Pol II directly and is recruited to early elongation complexes through DSIF. Although the details of the interaction/recruitment mechanism are still unclear, one possibility is that the negative elongation function of DSIF/NELF could be related to their ability to bind the nascent RNA. Particularly, NELF-E and Spt5 are both known to interact with the short RNA transcript emerging from Pol II after transcription initiation and thus inhibit productive elongation ^[21–23]^ (Figure 1). Crosslinking experiments suggest that Spt5 contacts the nascent RNA as it emerges from the elongation complex and subsequently recruits NELF ^[23]^. Both DSIF and NELF require a nascent RNA chain (>18 nt-long) to stably associate with the Pol II elongation machinery ^[23]^, in agreement with a lack of evidence for a role of DSIF/NELF in initiation and promoter clearance ^[20]^.

Using permanganate genomic footprinting, Lee *et al*. found that most genes marked by NELF in *Drosophila* also showed evidence of paused Pol II at promoter-proximal regions (+30 to +50 bp from the TSS) and proposed that NELF-mediated Pol II pausing might be an obligatory but sometimes transient checkpoint during the transcription cycle ^[24]^. Similarly, Rahl *et al*. has shown that NELF and DSIF co-occupy most Pol II regulated promoter-proximal regions of transcriptionally active but paused genes in mouse embryonic stem cells ^[12]^.

Although a subset of NELF target genes in *Drosophila* (like Hsp70) are up-regulated by NELF genetic depletion, the majority show decreased expression levels, suggesting that the presence of paused Pol II might enhance gene expression by maintaining a permissive chromatin architecture around the promoter-proximal region. Additionally, the loss of Pol II stalling at these promoters is accompanied by a significant increase in nucleosome occupancy and a decrease in the active chromatin signatures surrounding the TSS ^[25]^. In fact, in 2010 Gilchrist *et al*. provided compelling evidence for a key role of paused Pol II in competing with nucleosomes for occupancy of highly regulated promoters, thereby preventing the formation of repressive chromatin architecture to facilitate further or future gene activation ^[26]^.

Several genomic approaches in *Drosophila* and mammals have supported the model that NELF associates only with the 5′ end of genes, while DSIF travels with elongating Pol II since it is detected throughout the transcribing unit ^[9, 10, 15, 24, 25]^. For example, at the *Drosophila* hsp70 locus, following heat shock stimulation, DSIF and Pol II but not NELF were strongly recruited to chromosomal puffs harboring the hsp70 genes. Thus, both NELF and DSIF cause Pol II to pause in the promoter-proximal region, and the transcriptional activator heat shock factor (HSF) might cause NELF to dissociate from the elongation complex, and DSIF to travel with elongating Pol II.

Compared to the regulation in metazoan cells, the evidence for Pol II pausing is lacking in *S. cerevisiae*. NELF orthologues are conserved from *Drosophila* to humans, but absent in budding yeast ^[24]^, thus suggesting a more complex regulatory network as species evolved. In fact, phylogenetic analyses of components of the elongation control machinery indicate that the number of mechanisms utilized to regulate P-TEFb function increase as organisms show more complex developmental patterns ^[27]^.

Several additional factors are known to control Pol II pausing. For example, GDOWN1 (a substoichiometric Pol II subunit shown previously to render it responsive to Mediator) blocks the general transcription factor TFIIF and stabilizes paused Pol II at promoter-proximal regions of most human genes ^[28]^. There are several excellent reviews discussing the roles of GDOWN1 and other regulators in transcription elongation control. In this review article, we focus on the newly discovered PPM1G/PP2Cγ phosphatase ^[29,30]^ and its functional interplay with the early elongation machinery in the control of Pol II pausing and pause release.

## Transcriptional pause release

Promoter-proximal Pol II pausing is relieved by the action of positive transcription elongation factors. One of these key factors is the positive transcription elongation factor b (P-TEFb), a heterodimer of a cyclin subunit and cyclin-dependent kinase 9 (Cdk9). The regulatory cyclin (Cyc) subunit associated with Cdk9 is usually CycT1 or CycT2 and in some cases CycK ^[27, 31, 32]^. Cdk9 exists in two functional isoforms (a major 42 kDa form and a minor 55 kDa form) both of which associate with a cyclin subunit ^[33]^. P-TEFb phosphorylates the C-terminal domain (CTD) of the largest subunit of Pol II and negative elongation factors (DSIF and NELF) to promote the escape into productive elongation (also known as transcriptional pause release) ^[22, 27, 34, 35]^.

P-TEFb phosphorylates the Spt5 subunit of DSIF on C-terminal repeats (consensus = G-S-R/Q-T-P) to promote processive transcription elongation ^[35]^. With difference to the Pol II CTD residues phosphorylated by P-TEFb (Ser2 and Ser5), phosphorylation of Thr4 on the C-terminal repeats of Spt5 promote the elongation activity of DSIF. This phosphorylation event converts DSIF from a repressor to an activator and is thought to function in a manner analogous to the phosphorylated CTD (serving as an additional code for active elongation complexes) ^[35]^. Despite this remarkable discovery, it remains unclear how Spt5 phosphorylation contribute to the elongation process. It is likely that it may interact with different factors (protein and RNA) at promoter-proximal regions to relieve repressive associations with NELF or other negative factors in the early elongation complex.

In addition to DSIF and NELF, the primary target of P-TEFb is the Pol II CTD, which is an unusual polypeptide extension appended to the largest subunit (Rpb1). The CTD is inherently disordered and serves as a flexible binding scaffold for numerous protein interactions throughout the transcription cycle. In metazoans, the CTD comprises from 25 to 52 tandem copies of the consensus heptad repeat YSPTSPS. The repeats are post-translationally modified in almost every residue to orchestrate the association of different sets of factors with the transcript and influence the functional organization of the nucleus. Although recent in vitro studies have suggested that P-TEFb primarily targets Ser5 and Ser7 residues ^[36]^, in vivo studies showed that Ser2 phosphorylation is more strongly affected upon Cdk9 inhibition ^[18, 37]^. However, Cdk9 inhibition with DRB and flavopiridol can target several kinases, albeit with different affinities.

Despite the critical role of Cdk9 in the control of transcriptional pause release, other kinases (Cdk7, Cdk12, and Cdk13) have been implicated in CTD phosphorylation and the control of Pol II pause release and RNA processing. With the goal of keeping this review focused on the interplay between Cdk9 and the novel kinase regulator (PPM1G) here we include references to fundamental articles discussing roles of other CTD kinases in transcription control ^[38–41]^.

Taken together, mechanisms regulating Pol II pause release are vast and primarily rely on direct control of P-TEFb, which can be recruited to chromatin through interactions with different factors and probably in different catalytic states (active or primed) to phosphorylate its substrates at target genomic sites.

## Control of transcriptional pause release by the 7SK snRNP complex

**Figure 2:**
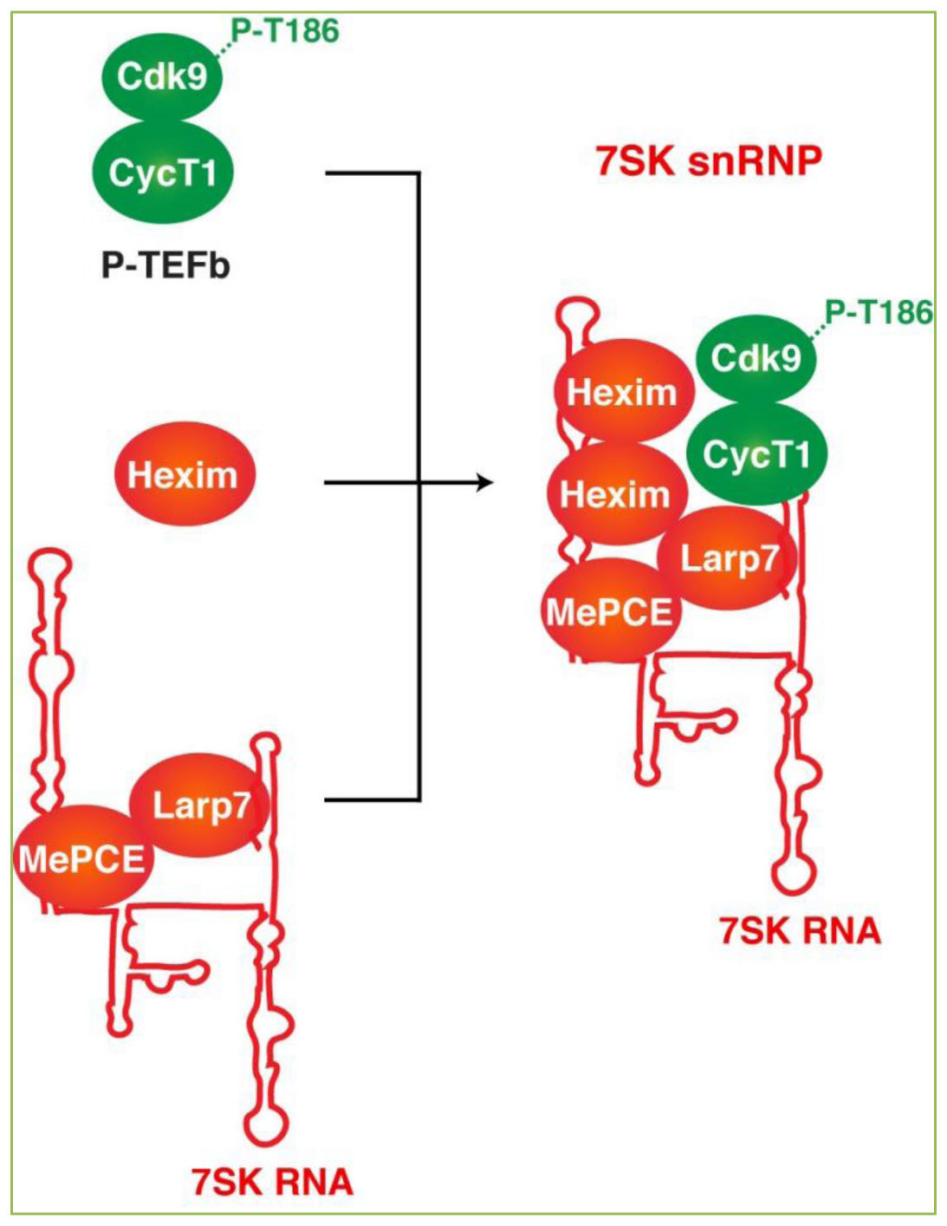
7SK snRNP complex assembly The 7SK snRNP complex assembles through interactions between P-TEFb (phosphorylated on the kinase T-loop, residue T186), the kinase inhibitor Hexim (that directly binds 7SK RNA as homodimer to tether the kinase), and MePCE-Larp7 proteins, which directly bind the RNA to protect it from degradation thus making a stable ribonucleoprotein complex.

Given the role of P-TEFb in pause release and the coupling between RNA processing and transcription elongation at most genes, mechanisms that control P-TEFb activity are of critical importance. These mechanisms include regulation of kinase expression, translation, and turnover ^[31, 42, 43]^. However, the most unique and well-studied mechanism of P-TEFb regulation is through the reversible association with the 7SK small nuclear ribonucleoprotein (snRNP) complex. In rapidly growing cells, a large fraction of P-TEFb (up to 90%) exists in a catalytically inactive form assembled into the 7SK snRNP ^[44]^, which is composed of the 7SK RNA, P-TEFb, the kinase inhibitor Hexim 1 and/or 2 (here referred as to Hexim for simplicity), the 5′-RNA methyl capping enzyme MePCE, and the 3′-RNA stability protein Larp7 ^[45–51]^ (Figure 2).

The human 7SK RNA is a 331 nt-long, abundant non-coding RNA synthesized by Pol III, and composed of four hairpins/stem loops (stems I, II, III and IV) ^[52]^. Previous reports have shown that MePCE interacts with the proximal-region of stem I and Larp7 binds to the U-rich tail region and other sites of the 3′-terminal stem IV ^[45, 47]^. MePCE and Larp7 form the core components of the 7SK snRNP and provide structural stability for the 7SK snRNA and the snRNP complex ^[47, 53, 54]^ (Figure 2).

The distal portion of 7SK stem I contains two structurally similar motifs, which function in the binding of Hexim1/2 homo- or heterodimers in vitro and in vivo ^[55, 56]^. The distal part of stems I and IV contain all necessary elements required for P-TEFb recruitment ^[57]^. Binding to 7SK RNA causes a conformational change in Hexim that allows it to interact with the CycT1 subunit allowing it to recruit P-TEFb, thus assembling the 7SK snRNP complex and inhibiting Cdk9 kinase activity ^[49, 58–61]^. Systematic 7SK RNA mutagenesis showed that alterations in Hexim binding abolishes P-TEFb’s recruitment thereby indicating its absolute requirement in formation of the 7SK snRNP ^[57]^. Moreover, 7SK snRNP assembly requires phosphorylation of the Cdk9 T-loop (at residue T186), which promotes interactions with Hexim and 7SK RNA in vitro ^[48]^.

In Hexim, two coiled-coiled regions [CCR1 (279–315) and CCR2 (319–352) in the C-terminus] and a basic RNA-binding motif (KKKHRRRP) that contacts 7SK RNA in a P-TEFb independent manner are involved in assembling the 7SK snRNP complex to inactivate the kinase ^[57]^. The CCR2 motif mediates Hexim homodimerization resulting in the recruitment of P-TEFb through the CCR1 region ^[58, 59]^. Although the molecular details for the protein-RNA interactions are well established, the lack of a high-resolution structure so far impedes the better understanding of the assembly and kinase inactivation processes.

Although the majority of the 7SK snRNP complex exists in the soluble nucleoplasmic fraction, previous studies have shown that the snRNP is recruited to promoter-proximal regions ^[29, 62–64]^, probably to deliver primed kinase for ‘on site’ activation ^[65]^. These discoveries were initially seen to be controversial in the field because of the low crosslinking efficiency of 7SK snRNP components compared to factors directly bound to chromatin. Particularly because, biochemically, 7SK snRNP appears not to be stably associated with chromatin and can freely diffuse in the nucleus, as demonstrated by its extraction using mild conditions (low detergent and salt concentrations) ^[44, 66]^. However, several studies by our group and others have extended those initial discoveries by providing compelling evidence that components of the 7SK snRNP complex are recruited to genomic domains including promoters and cellular enhancers to control transcription elongation ^[29, 64, 65, 67]^. Thus, the presence of P-TEFb as part of the 7SK snRNP complex at gene promoters correlates with the degree to which Pol II pauses probably indicating a direct role in positively controlling transcription elongation ^[29, 63–65]^. In this context, the 7SK snRNP complex appears not to simply be an inactive reservoir of P-TEFb but plays a functional role in delivering primed kinase to promoter-proximal regions containing paused Pol II.

## Interplay between the 7SK snRNP, P-TEFb and cellular phosphatases in regulating transcriptional pause release

**Figure 3:**
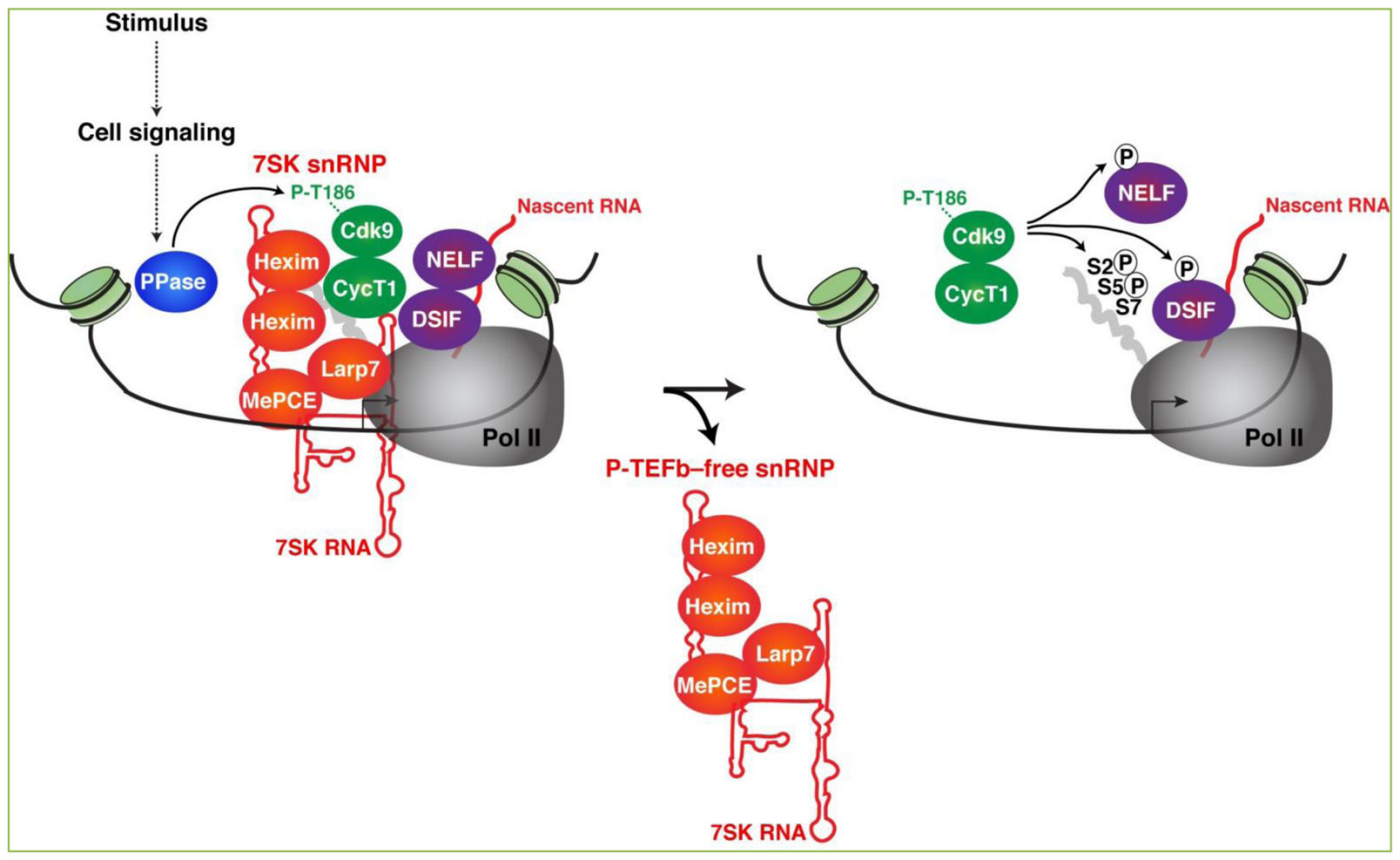
P-TEFb dissociation from the 7SK snRNP by the action of phosphatases promotes transcriptional pause release In response to intrinsic/extrinsic stimulus, cell-signaling cascades are activated, and phosphatases (PPase) are induced to promote Cdk9 T-loop dephosphorylation (P-T186) causing P-TEFb release from the 7SK snRNP complex. However, it remains unclear whether the PPases function on or off chromatin (or both). Released P-TEFb can then phosphorylate the Pol II CTD on Ser5 (S5) and Ser2 (S2) residues, DSIF (which is converted into a positive elongation factor), and NELF (which is evicted from Pol II) thereby relieving Pol II stalling at promoter-proximal regions. Upon 7SK snRNP disassembly, the P-TEFb free snRNP is repositioned in the promoter-proximal region or dislodged from chromatin. This aspect of the model requires further investigation (see the text for details). The arrow indicates the position of the TSS.

In order to accommodate the transcriptional needs of the cell, the P-TEFb kinase has to be released from the snRNP and then captured by promoter-associated factors to act locally on its substrates (Pol II and negative elongation factors). Just as Cdk9 T-loop phosphorylation promotes 7SK snRNP complex assembly, dephosphorylation of the T-loop by phosphatases (PPases) induces P-TEFb dislodgement from Hexim thereby causing 7SK snRNP disassembly and transcriptional pause release (Figure 3). While several phosphatases have been identified and proposed to function through the 7SK snRNP, the mechanisms (in vitro and in vivo) have not been closely examined. In 2008, two papers described phosphatases of different families as substrates of the P-TEFb kinase ^[68, 69]^. The first Ser/Thr phosphatases to be functionally linked to P-TEFb activity control through the 7SK snRNP were PP2B and PP1α ^[68]^, members of the PPP phosphoprotein family of phosphatases ^[70]^. Both enzymes were proposed to cooperatively disrupt the 7SK snRNP complex to release P-TEFb for transcription in response to Ca2+ signaling ^[68]^.

Another phosphatase is PPM1A/PP2Cα, which is a member of a large family of metal-dependent phosphatases (PPM/PP2C) that participates in diverse biological processes ^[71]^. PPM1A overexpression in cells induces dephosphorylation of the Cdk9 T-loop ^[69]^. However, the biological relevance of this regulatory step in the control of P-TEFb activity and Pol II transcriptional pause release, as well as the target genes regulated remains to be elucidated.

**Figure 4:**
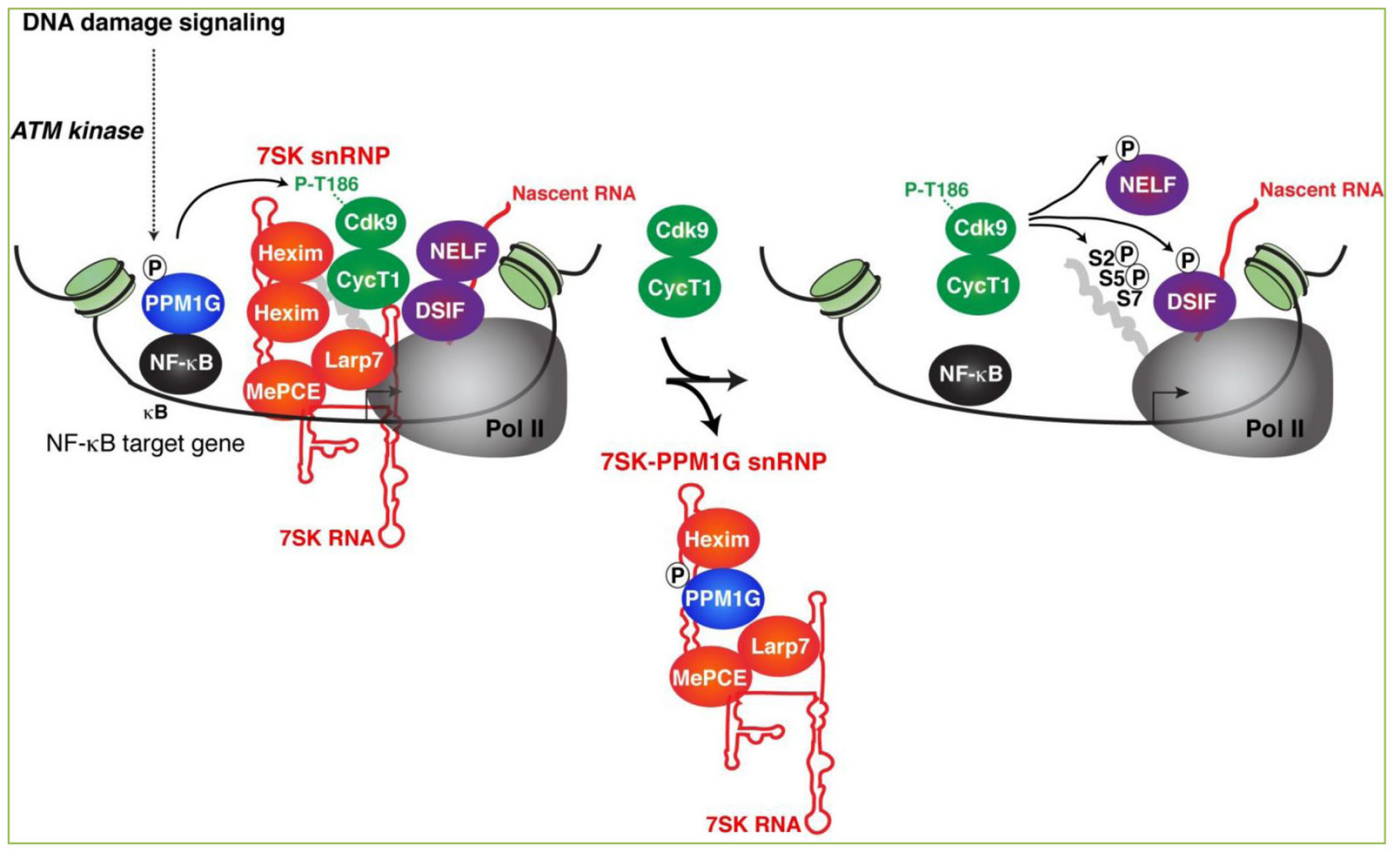
PPM1G functions through the promoter-assembled 7SK snRNP complex to induce Pol II elongation in response to DNA damage signaling In response to DNA damage signaling, ATM activates the NF-κB signaling pathway (NF-κB binds to target gene promoters) and concomitantly phosphorylates PPM1G (P), which in turn dephosphorylates and releases the P-TEFb kinase from the promoter-bound 7SK snRNP complex. Upon its release, dephosphorylated P-TEFb becomes re-phosphorylated and activated, and thus acts on paused Pol II to induce the transcriptional pause release. After releasing P-TEFb, the inhibitory snRNP subunits are evicted from chromatin, and phosphorylated PPM1G (P-S183) binds 7SK RNA along with Hexim (7SK-PPM1G snRNP) to prevent the re-association of P-TEFb back into the snRNP to sustain transcription elongation. Once the damage is resolved, this regulatory circuitry subsides, PPM1G is dislodged from the snRNP (through a yet unknown mechanism), and P-TEFb recycled back to promote the formation of the inhibitory 7SK snRNP at the promoter-proximal region, thereby blocking Pol II pause release. The arrow indicates the position of the TSS.

More recently, using a biochemical approach, McNamara *et al*. identified another PPM/PP2C family member (PPM1G/PP2Cγ) that dephosphorylates the T-loop of Cdk9 to promote the disassembly of the 7SK snRNP complex in vitro (Figure 4). More importantly, PPM1G is recruited to gene promoters in response to a variety of environmental signals (inflammatory stimuli or DNA damage) through interaction with the master transcriptional regulator NF-κB ^[29,30]^. The study by McNamara *et al*. represents the first set of findings demonstrating a functional link between the recruitment of a Cdk9 T-loop phosphatase, 7SK snRNP disassembly, and activation of transcriptional pause release in response to stimulation in vivo ^[29]^. This report also exemplifies a mechanism through which P-TEFb can be activated to induce Pol II pause release at a subset of genes in response to stimulation.

The dephosphorylation of Cdk9 T-loop poses a problem with the kinase activation state because Cdk9 has to be phosphorylated on the T-loop to become active in addition to requiring a conformational change in the cyclin subunit ^[48,72,73]^. Two different mechanisms have been proposed to phosphorylate the T-loop. The first mechanism is through Cdk7, which is the TFIIH kinase of the transcription pre-initiation complex (PIC) (see Figure 1). Interestingly, Cdk7 inhibition increases initiation factor (TFIIE) retention at the promoter, prevents DSIF recruitment and attenuates Pol II pausing and pause release, due to a reduction in Pol II CTD Ser2 phosphorylation and H2B ubiquitylation ^[41]^. The second mechanism implies kinase autophosphorylation induced by the interaction between P-TEFb and transcription factors (probably involving major conformational changes) ^[29]^. In this context, protein interactions causing the induced fit of HIV Tat on P-TEFb exemplifies that transcription factors can promote conformational changes on P-TEFb and regulate its kinase activity ^[73, 74]^. These are two possible mechanisms that describe how P-TEFb (released from the 7SK snRNP complex) can undergo rapid activation through T-loop rephosphorylation (Figure 4).

Together, this underscores the importance of 7SK snRNP’s recruitment to chromatin and its interplay with cellular phosphatases (like PPM1G) to facilitate Pol II pause release of target genes in response to environmental stimulation.

## Role of PPM1G in relieving transcriptional pausing through the 7SK snRNP

While low levels of PPM1G are detected at promoter-proximal regions of inducible or primary responsive genes (such as those in the NF-κB network), its occupancy robustly and temporally increases in response to DNA damage stimulation as revealed by kinetic ChIP assays ^[30]^. In response to DNA damage signaling, PPM1G occupancy levels at NF-κB target gene promoters rises in parallel to the increase in P-TEFb/7SK snRNP. At these sites, PPM1G dephosphorylates the T-loop of Cdk9, which uncouples it from the kinase inhibitor Hexim, thereby causing disassembly of the 7SK snRNP complex, and inducing P-TEFb activation and Pol II pause release ^[29,30]^ (Figure 4). Interestingly, following the release of P-TEFb from the 7SK snRNP, we found that PPM1G directly binds 7SK RNA and forms a heterodimer with Hexim (henceforth referred to as “7SK-PPM1G snRNP” to differentiate it from the P-TEFb bound 7SK snRNP complex containing all subunits). This mechanism serves to block formation of the inhibitory 7SK snRNP complex increasing P-TEFb’s availability to participate in multiple rounds of elongation (without being sequestered back by Hexim into the 7SK snRNP). The PPM1G-7SK RNA interaction is direct, kinetically follows the recruitment of PPM1G to target gene promoters, and temporally precedes transcription activation, supporting its role as a transcriptional co-activator. The interaction between PPM1G and 7SK RNA is reversible, since PPM1G dissociates from the RNA upon resolution of the transcriptional program, thereby allowing for the re-sequestration of P-TEFb and Hexim into the snRNP and resetting the system to the basal, repressed state, thus terminating transcription elongation programs ^[30]^. Thus, the reversible nature of the PPM1G-7SK snRNP interaction facilitates both the initiation and termination of the elongation programs. Together, PPM1G provides a functional link between Cdk9 T-loop dephosphorylation and, both activation and maintenance of the active transcriptional state (an unprecedented regulatory step in transcriptional regulation).

PPM1G is composed of three discrete domains: N-terminal, C-terminal phosphatase domain containing a Lysine-rich region (Lys), and an intervening acidic-rich region. PPM1G uses the Lys-rich region, which bears similarity to the Hexim RNA-binding motif ^[56]^, to interact with the distal region of 7SK RNA stem I. PPM1G binding is in close-proximity to the Hexim-binding site, and blocks the CCR1 site on Hexim (the site of P-TEFb interaction), which is all consistent with formation of Hexim-PPM1G-7SK RNA complexes ^[30, 49, 57, 75]^. Together, this suggests that assembly of the PPM1G-Hexim protein complex on 7SK RNA functions to block P-TEFb binding ^[30]^.

Several heterogeneous nuclear ribonucleoproteins (hnRNP) such as hnRNP A1, A2/B1, R and Q have been previously shown to associate with 7SK RNA, but only after Hexim and P-TEFb have dissociated from the RNA ^[76, 77]^. It would be interesting to determine whether these factors function in an analogous manner to PPM1G or whether they cooperate with PPM1G and Hexim in preventing P-TEFb association into the 7SK snRNP complex to sustain transcription elongation.

## PPM1G is required for normal biological processes and could prevent malignancy

The critical role that PPM1G plays in the transcriptional cycle and in the control of cell fate choice indicates that its malfunction can promote malignancy. Given that DNA damage activates the NF-κB signaling pathway and that PPM1G is a transcriptional co-activator of NF-κB, its function is essential in maintaining cellular homeostasis. In response to DNA damage, PPM1G regulates NF-κB mediated 7SK RNA dependent Pol II transcription ^[30]^. Consistent with the role of the ataxia telangiectasia mutated (ATM) kinase in regulating DNA damage signaling, we found that ATM regulates the interaction between PPM1G and the 7SK snRNP (by enhancing 7SK RNA interaction) through PPM1G site-specific phosphorylation. Due to the critical role PPM1G plays in transcription and activation of intrinsic and extrinsic pathways it would be interesting to determine if cancer-related mutations in the phosphatase could act as drivers of malignancy or remain passenger genetic alterations. Furthermore, if activation of key cellular programs is required to elicit critical responses to a DNA insult then it is possible that PPM1G would function as tumor suppressor. Honing our understanding about the roles PPM1G plays as transcriptional co-activator and in preventing the onset of malignancy become critical points as we move forward in defining the functional interplay between this enzyme and the transcription elongation machinery.

Since PPM1G participates in the activation of NF-κB programs and NF-κB is a critical tumor suppressor or oncogene (depending on the stimuli, cell-type, and context ^[78]^), PPM1G also emerges as a critical target for diseases where NF-κB is constitutively activated (such as in chronic inflammatory disorders, skin cancers, and leukemias, among others).

## Conclusions and future challenges

A common theme in the field is the identification of signaling components/factors that promote the release of P-TEFb from the 7SK snRNP to relieve transcriptional pausing in what seems to be a highly intricate and dynamic process. Although several factors have been linked to transcriptional pause release control, little is known about how these factors interact with paused Pol II and the chromatin environment at promoter-proximal regions. Also, it remains unclear what is the role of the transcription factors in linking co-activators to promoters to facilitate pause release. Some co-activators functioning to promote transcription elongation through the 7SK snRNP complex have been recently identified, such as the JMJD6 histone demethylase, the bromodomain-containing protein BRD4, SR splicing factors, and the RNA helicase DDX21 ^[64, 67, 79]^. Nonetheless, PPM1G provides the first example of a cellular enzyme that functions through the non-coding RNA to both activate and maintain transcription elongation programs ^[30]^. PPM1G, compared to other phosphatases described above, is the only known phosphatase that has been shown to associate with 7SK RNA or other snRNP components to control assembly of the 7SK snRNP complex. However, the role of other phosphatases cannot be overlooked, as it still remains unclear if PPM1G is used as a general transcriptional co-activator or only by a subset of P-TEFb regulated genes or under specific regulatory conditions. It is possible that other phosphatases could regulate 7SK snRNP dissociation and P-TEFb release for transcription elongation under different environmental conditions or at different target genes.

Despite the recent remarkable discoveries in the field, several critical points remain to be answered. Why P-TEFb/7SK snRNP is targeted to promoters and enhancers? What is the benefit of having P-TEFb assembled at these genomic sites? What does control the timing of kinase activation where the substrates are present? How does PPM1G encounter its substrate (Cdk9) as part of a larger 7SK snRNP complex? Furthermore, although the molecular and structural details of the interaction between PPM1G and 7SK snRNP provided important insights in the control of transcription elongation, several key questions remain for the future. For example: Do posttranslational modifications keep the Hexim-PPM1G complex bound to the snRNP? And if yes, which enzymes regulate this process? How does PPM1G dissociate from Hexim and 7SK RNA to allow re-association of P-TEFb and inactivate Pol II pause release?

Since PPM1G interacts with other snRNP components such as MePCE and Larp7 ^[29]^, in vitro studies defining the stoichiometry and molecular nature of the interactions would be beneficial to understand the assembly mechanism and to pave the way for future functional studies. Finally, due to the critical role of negative elongation factors (NELF and DSIF) in the control of Pol II pausing/pause release, whether PPM1G enzymatically targets these and other elongation factors while preventing escape into productive elongation remains to be investigated. In sum, understanding the intricate functional interplay between PPM1G, the 7SK snRNP, and other transcription co-factors are future challenges for the field.

## Abbreviations

ATM: ataxia telangiectasia mutated
DRB: 5,6-dichloro-1-β-D-ribofuranosyl-benzimidazole
DSIF: DRB sensitivity-inducing factor
CCR: coiled-coil region
Cdk9: cyclin-dependent kinase 9
Cyc: cyclin
CTD: C-terminal domain
hnRNP: heterogeneous nuclear ribonucleoprotein
HSF: heat shock factor
Lys: Lysine-rich region
NELF: negative elongation factor
P-TEFb: positive transcription elongation factor b
PIC: transcription pre-initiation complex
Pol II: RNA polymerase II
PPase: protein phosphatase
snRNP: small nuclear ribonucleoprotein
TSS: transcription start site
